# Cultural techniques capture diverse phosphate-solubilizing bacteria in rock phosphate-enriched habitats

**DOI:** 10.3389/fmicb.2024.1280848

**Published:** 2024-02-07

**Authors:** Amandine Ducousso-Détrez, Zakaria Lahrach, Joël Fontaine, Anissa Lounès-Hadj Sahraoui, Mohamed Hijri

**Affiliations:** ^1^Département de Sciences Biologiques, Institut de Recherche en Biologie Végétale (IRBV), Université de Montréal, Montréal, QC, Canada; ^2^Université du Littoral Côte d’Opale, UR, Unité de Chimie Environnementale et Interactions sur le Vivant (UCEIV), SFR Condorcet FR CNRS, Calais, France; ^3^African Genome Center, University Mohammed VI Polytechnic (UM6P), Ben Guerir, Morocco

**Keywords:** rock phosphate, phosphate-solubilizing bacteria, bioinoculants, rhizoplane, hyphosphere, mining area

## Abstract

Phosphorus (P) deficiency is a common problem in croplands where phosphate-based fertilizers are regularly used to maintain bioavailable P for plants. However, due to their limited mobility in the soil, there has been an increased interest in microorganisms that can convert insoluble P into a bioavailable form, and their use to develop phosphate-solubilizing bioinoculants as an alternative to the conventional use of P fertilizers. In this study, we proposed two independent experiments and explored two entirely different habitats to trap phosphate-solubilizing bacteria (PSBs). In the first experiment, PSBs were isolated from the rhizoplane of native plant species grown in a rock-phosphate (RP) mining area. A subset of 24 bacterial isolates from 210 rhizoplane morphotypes was selected for the inorganic phosphate solubilizing activities using tricalcium phosphate (TCP) as the sole P source. In the second experiment, we proposed an innovative experimental setup to select mycohyphospheric bacteria associated to arbuscular mycorrhizal fungal hyphae, indigenous of soils where agronomic plant have been grown and trapped in membrane bag filled with RP. A subset of 25 bacterial isolates from 44 mycohyphospheric morphotypes was tested for P solubilizing activities. These two bacterial subsets were then screened for additional plant growth-promoting (PGP) traits, and 16S rDNA sequencing was performed for their identification. Overall, the two isolation experiments resulted in diverse phylogenetic affiliations of the PSB collection, showing only 4 genera (24%) and 5 species (17%) shared between the two communities, thus underlining the value of the two protocols, including the innovative mycohyphospheric isolate selection method, for selecting a greater biodiversity of cultivable PSB. All the rhizoplane and mycohyphospheric PSB were positive for ammonia production. Indol-3-acetic acid (IAA) production was observed for 13 and 20 isolates, respectively among rhizoplane and mycohyphospheric PSB, ranging, respectively, from 32.52 to 330.27 μg mL^−1^ and from 41.4 to 963.9 μg mL^−1^. Only five rhizoplane and 12 mycohyphospheric isolates were positively screened for N_2_ fixation. Four rhizoplane PSB were identified as siderophore producers, while none of the mycohyphospheric isolates were. The phenotype of one PSB rhizoplane isolate, assigned to *Pseudomonas*, showed four additive PGP activities. Some bacterial strains belonging to the dominant genera *Bacillus* and *Pseudomonas* could be considered potential candidates for further formulation of biofertilizer in order to develop bioinoculant consortia that promote plant P nutrition and growth in RP-enriched soils.

## Introduction

Agricultural production in conventional cultural systems relies on continual inputs of chemical P-fertilizers. However, their industrial production and processing is costly and can lead to environmental concerns if applied in excess (Hudson-Edwards, 2016). Therefore, to reduce the environmental footprint of agricultural practices, sustainable alternatives that reduce over-reliance on chemical fertilizer applications while maintaining crop production, have been tested (Shen et al., 2011). Studies have examined the various functions of microbial sources found in the root environment to produce microbial-based agricultural inputs which are effective in improving plant growth with reduced mineral intake (Compant et al., 2019; Elhaissoufi et al., 2022). Therefore, Plant Growth-Promoting Rhizobacteria (PGPR), bacteria that promote plant growth, have become the focus of attention of researchers (Richardson et al., 2009; Vacheron et al., 2013). Particularly, phosphate-solubilizing bacteria (PSB) have been proposed as a feasible solution to ameliorate the bioavailable-phosphate deficiency in cropping systems. These bacteria convert insoluble and organic P compounds into soluble and bioavailable forms (H_2_PO_4_^−^ and HPO_4_^2−^) with the aid of organic acids and enzymes such as phosphatases and phytases (Goldstein, 1995; Ahmed et al., 2021). This, in turn, allows them to benefit from P while also enabling other organisms, such as plants, to access it as a nutrient source (Raymond et al., 2021). As such, these bacteria have been highlighted as key players in the biogeochemical P cycle (Tian et al., 2021; Ducousso-Detrez et al., 2022a,b). Thus, the development of phosphate-solubilizing bio-inoculants has been suggested as an alternative to the regular use of P fertilizers. Moreover, some particular PGPR/PSB strains have been shown to possess several growth-promoting properties, such as the production of hormones, antibiotics, and enzymes to enhance plant growth and protection against pathogen attacks (Kour et al., 2021; Alotaibi et al., 2022). An example of this was demonstrated by *Aneurinibacillus aneurinilyticus* CKMV1, which was shown to possess several plant growth-promoting activities, including N-fixation, indole-3-acetic acid production, siderophore synthesis, hydrogen cyanide production, and antifungal activity (Chauhan et al., 2017). Siderophore production is a crucial factor for promoting plant growth. Indeed, siderophores are high-affinity iron-chelating compounds that can acquire ferric Fe^3+^ from mineral phases, scavenging it to make it available as the preferred form of Fe^2+^ for uptake by plant roots (Vessey, 2003).

Furthermore, the use of microbial and mineral P resources together has been gaining more attention as a potential solution. When used in combination, they may act synergistically to increase the agronomic efficiency of mineral fertilizers and supply the essential nutrients and functions needed for plant growth and development. Notably, the combined use of PSB-based inoculants and rock phosphate (RP) has led to a successful “microbial-P mineral alliance” (Bargaz et al., 2018; Tahir et al., 2018). Indeed, RP is a geological P-rich rock used alone in some agricultural systems to efficiently improve crop production with lower costs and environmental damage than water-soluble P chemical fertilizers (Sharma et al., 2013; Ahemad and Kibret, 2014). However, due to its low solubility in water (largely linked to its chemical, crystallographic, and mineralogical composition of its apatites), RP reactivity (i.e., the rate of orthophosphate ion release when applied directly to soils under favorable soil conditions) and its agronomic effectiveness are generally lower than those of commercial fertilizers (Verma et al., 2012). Nevertheless, a number of studies have evidenced improved P availability in soils when RP is combined with PSB (Bargaz et al., 2018; Elhaissoufi et al., 2022), with significant increases in P uptake, shoot/root biomass, and yield performances compared to inoculation treatments used singly (Kaur and Reddy, 2015; Manzoor et al., 2016; Adnan et al., 2017; Ditta et al., 2018; Elhaissoufi et al., 2020).

The identification of PSB has thus become a relevant research field for the development of a more sustainable farming system that benefits the environment and society, and increases farmers’ income (Verma et al., 2015; Lobo et al., 2019). Here, the literature clearly shows that numerous commercial PSB strains and bioinoculants including PSB strains are already available on the global market (Owen et al., 2015; Koskey et al., 2021). However, the success of microbial inoculation is also open to debate (Basiru and Hijri, 2022). Indeed, as its effectiveness is highly dependent on environmental conditions (i.e., strongly constrained by soil properties, and indigenous microbial populations in particular). To face such a challenge, a larger diversity in the set of cultivable PSB isolates could provide a response to the soil diversity in which inoculants will be used. It could also open new ways for better mechanistic understanding of the PSB phenotype.

Based on this hypothesis, new PSB identification therefore required new trapping strategies, based for instance on new media for *in vitro* selection and culture of isolates. It is also crucial in promoting a diversification of sampling environments and habitats, each inhabited by its own microbiome, leading potentially to isolates not yet identified.

Screenings for PSB are typically performed from rhizospheric soils or roots, where it is widely accepted that PSB are abundant (Ashok et al., 2012; Baliah et al., 2016; Alotaibi et al., 2022). PSB have also been screened from the soil environment along the extraradical hyphae of arbuscular mycorrhizal fungi (AMF): the mycohyphosphere (Johansson et al., 2004; Toljander et al., 2006; Lecomte et al., 2011). In this microhabitat, it is known that some bacteria can adhere to the surface of AMF hyphae (Ordonez et al., 2016; Taktek et al., 2017). Nonetheless, the identity and potential roles of microbes associated with AMF hyphae, along with the mechanisms enabling the recruitment and cooperation of beneficial microbes, remain inadequately understood. However, there is evidence indicating that bacteria associated with AMF can exhibit diverse functional traits, such as nitrogen fixation, phosphate mobilization, growth hormone production, biofilm production, and biocontrol against pathogens (Barea et al., 2002; Frey-Klett et al., 2007; Ujvári et al., 2021). These traits may play a significant role in maintaining the health of AMF symbiosis and fostering plant growth [reviewed in Basiru et al. (2023) and Wang L. et al. (2023)]. Thus, Cruz and Ishii (2011) successfully isolated probable endobacteria such as *Bacillus* and *Paenibacillus* isolates from *Gigaspora margarita* spores that exhibited multiple PGP traits, including P solubilization. Similarly, Battini et al. (2017) showed positive effects of AMF and their associative endobacteria isolated from AMF spores, regarding facilitation of P uptake under P-limiting conditions. Benefits also occur for AMF and hyphae-associated PSB communities, as interactions provide key resources to each other (Zhang et al., 2016). In particular, AMF hyphae exudates (sugars, amino acids, carboxylates) may provide key nutrients for bacterial growth, while attachment of bacteria with P solubilizing capacity to the extraradical AMF hyphae can allow the AMF to get additional soluble orthophosphate ions (Jansa et al., 2013; Zhang et al., 2018; Jiang et al., 2021). Additionally, PSB can access nutrients with limited diffusion in soils, inside the mycorrhizosphere (Ordonez et al., 2016). As a result, there is a growing interest in exploring the diversity and functions of bacteria associated with AMF hyphae (Toljander et al., 2007; Faghihinia et al., 2022; Basiru et al., 2023). Speculations regarding potential positive interactions between PSB and AMF in nutrient acquisition have increased, making the isolation of mycohyphospheric PSB a promising avenue for developing formulations of biofertilizer inoculants (Jansa et al., 2013; Zhang et al., 2016).

Consequently, in this study, we proposed two independent experiments and explore two entirely different habitats to trap PSB isolates in RP-rich soils. In the first experiment the PSBs were isolated from the rhizoplane of native plant species growing in a rock-phosphate mining area. In the second experiment, we proposed an innovative experimental setup to trap mycohyphospheric bacteria associated to arbuscular mycorrhizal fungal hyphae, indigenous of soils where agronomic plant have been grown. The selected PSB were further screened for additional conventional plant growth-promoting traits, and the isolates were identified by 16S rRNA sequencing and phylogenetic analysis. The taxonomic and functional diversity of the selected isolates were compared and discussed as a potential component in further inoculant formulations for increasing crop production, especially in RP-enriched soils.

## Materials and methods

### Experimental setup for isolation of rhizoplane bacteria

#### Sampling sites

In order to isolate PSB from the rhizoplane, samplings were performed in a former RP mining area located in the National Nature Reserve of Geological Interest of the Lot department in France (44° 22′ 22″ N, 1° 41′ 16″ E). Three locations (L1: 44°21′03,70″N; 1°41′26,66″E – L2: 44°21′44,65”N; 1°41′16,02″E – and L3: 44°29′15,85”N; 1°48′13,19″E) were identified, each providing two sites characterized by contrasting P concentrations due to mining activities in the past (high P and low P). Indeed, during mining operations, the most RP-enriched soil fractions were exported and used to manufacture P fertilizers, while the finer mine tailings were left in place. This resulted in surface soils that were locally enriched in phosphate (hereinafter referred to as P soils) side by side with soils not enriched in phosphate (nP soils).

In each site, the soil physicochemical properties, including pH, soil texture, and chemical composition, were assessed by the CIRAD-US Analyse laboratory in Montpellier, France, utilizing inductively coupled plasma spectrometry, atomic emission spectrometry, and X-ray fluorometry (Ducousso-Detrez et al., 2022b). The six soils exhibited near-neutral pH levels, ranging between 6.9 and 7.3. Regarding granulometry, all topsoil (0–30 cm) exhibited elevated levels of clay, with additional higher percentages of coarse sands observed in P soils.

In nP soils, total P concentrations ranged from 1,057.1 to 1,496.3 mg/kg, while in P soils, they varied from 2,880.0 to 13,927.9 mg/kg. Similarly, available Olsen P concentrations in P soils ranged from 46.1 mg/kg to 339.5 mg/kg, while nP soils were characterized by values ranging from 5.04 to 12.82 mg/kg. Through the comparison of matched soils from the same location, P and Olsen P ratios, ranging from 2.07 to 13.05 and from 9.14 to 41.96 Pi, respectively, were derived. These ratios allowed for the classification of P sites as high-P sites and nP sites as low-P sites.

#### Plant sampling

**Figure 1:**
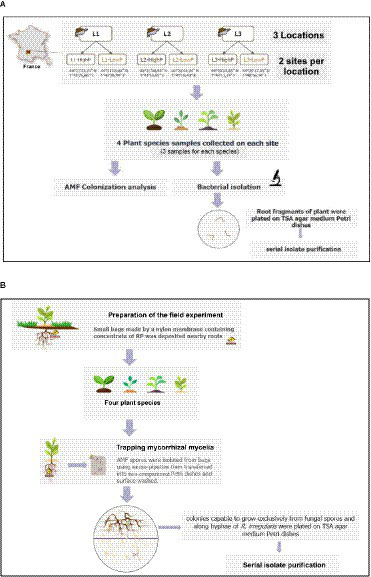
Experimental setup for root **(A)** and mycohyphosphere **(B)** bacteria isolation.

In January 2019, samples of native plant species (*Bromus sterilis, Dactylis glomerata, Taraxacum officinalis,* and *Ranunculus bulbosus*) were collected from various mining sites. For each of the six sites surveyed, three individual plants per plant species were collected, resulting in the harvest of a total of 72 plants. These plants were carefully placed in sterile plastic Whirl-Pack bags and transported to the laboratory with the aid of an ice pack. Subsequently, the roots were separated from the aerial parts, and the soil adhering to roots was eliminated by vigorously shaking the roots. Fresh root segments were washed by shaking in 90 mL of sterile saline solution (NaCl, 0.85% W/V) before to be plated on Tryptic Soy Agar (TSA) agar medium Petri dishes. Colonies were picked up after one week of culture and spread over TSA nutrient plates. Serial subcultures were then performed on the same medium and, if required, isolate purification was conducted (Figure 1A).

### Experimental setup for isolation of mycohyphospheric bacteria

Mycohyphospheric bacterial isolates were selected through an experimental process consisting of three steps. The first was conducted with the aim of trapping mycorrhizal structures (spores and hyphae) of native AMF. It was conducted at the Montreal Botanical Garden (Montreal, QC, Canada) in June 2018, in a plot with a Black Chernozem soil used exclusively for organic farming practices. Small bags (5 cm width x 10 cm length) made of a nylon membrane (pore size of 200 μm) containing igneous RP (with 54% of P_2_O_5_, Quebec, Canada) or sedimentary RP (with 39% of P_2_O_5_, Morocco) were placed near the roots of potato, tomato, leek, and maize plants. After three months, the RP bags were removed from the soil. A supplementary step was conducted to extend the time required for spore development and increase the number of spores inside the RP bags. It was carried out in a greenhouse (22°C with a photoperiod of 8-16 h) with RP bags placed near the roots of plants grown in pots. The soil substrate was watered to field capacity with tap water as needed and fertilized weekly with a Hoagland solution without P. After six months, bags were collected from each pot. The contents of each bag were placed on a Petri dish, covered with sterile distilled water. Subsequently, utilizing gentle and repetitive circular motions, hyphae with attached spores were gathered around a glass Pasteur pipette and transferred into another Petri dish. The spores/hyphae mixture underwent successive washes with sterile water containing EDTA (0.5%). Finaly, under a stereomicroscope, spores were individually collected using a 20 μm micropipette and transferred into a microtube containing sterile distilled water. In these conditions, 30–40 spores per 10 g of soil were successfully obtained.

The third step of the experimental setup aimed to isolate bacterial strains from AMF spores previously trapped and harvested from the RP bags. It was performed *in vitro*, using a two-compartment Petri dish design, as previously described by St-Arnaud et al. (1995) then modified by Taktek et al. (2017). This design was used to isolate mycohyphospheric bacteria able to grow on the surface of AMF hyphae, utilizing the extraradical mycelium exudates as a sole source of energy. Thus, the AMF *Rhizophagus irregularis* DAOM 197198 (originally isolated from Pont-Rouge, QC, Canada) was grown on *Agrobacterium rhizogenes*-transformed carrot (*Daucus carota* L.) roots (IRBV, QC, Canada), in the first Petri dish compartment filled with 20 mL of M medium (Becard and Fortin, 1988). The second compartment received 20 mL of M medium without any carbon source or vitamins and was kept root-free by trimming the roots to exclusively facilitate the growth of AMF extraradical hyphae. Both media were solidified with 0.4% (W/V) of Phytagel (Sigma-Aldrich, Oakville, ON, Canada). The plates were incubated for approximately 5 weeks at 25°C in the dark until the hyphae colonized the second compartment. The spores, which had been previously confined within RP bags and manually retrieved through microscopic observations, were subsequently placed onto the *R. irregularis* hyphae growing in the distal compartment. The Petri dishes were then incubated for 5 days at 25°C and observed daily to check the viability of hyphae. The bacterial colonies capable of growing along the *R. irregularis* hyphae, without any visible damage, were isolated and reinoculated repeatedly until single morphotypes were obtained on 10% TSA (QueLab, QC, Canada). Hereafter, they were referred to as mycohyphospheric bacteria (Figure 1B).

### *In vitro* screening for inorganic phosphate solubilizing bacteria

After serial purification, the rhizoplane and mycohyphospheric bacterial isolates were spread on modified National Botanical Research Institute’s Phosphate (NBRIP) agar medium, containing tricalcium phosphate (TCP) (Nautiyal, 1999). NBRIP plates were incubated at 28°C for 14 days and colonies forming a clear solubilization halo, which results from their TCP solubilizing activity, were taken as PSB.

The phosphomolybdenum blue method was performed to quantitatively assess the P-solubilization capabilities of isolates in a solution. In this method, P ions react with acid ammonium molybdate to generate phosphomolybdenum complexes. These complexes are then reduced by ascorbic acid, leading to the formation of a highly pigmented phosphomolybdenum blue species. The degree of blue coloration, measured spectrophotometrically at 820 nm, correlates proportionally with the phosphate concentration (Howe and Mellon, 1940; Aliyat et al., 2020). Thus, NBRIP medium in a 250 mL flask was inoculated with an aliquot of an overnight bacterial culture (OD 600 nm = 0.7) and incubated under shaking conditions for 7 days at 28°C. After centrifugation (for 10 min, 12,000 g), 0.5 mL of supernatant was initially added to trichloroacetic acid (10% W/V), followed by a mixture containing ammonium molybdate and ascorbic acid in a sulfuric acid solution. The flask was then placed in the dark for the color reaction. The quantitative estimation of P solubilization in the bacterial culture supernatant was determined against a KH_2_PO_4_ standard curve.

### Culture preservation and maintenance

The selected PSB isolates were stored in 25% glycerol at −80°C. After preservation, all subcultures were performed in 50 mL of 10% TSA medium (Difco Laboratories Inc. Detroit, MI, USA) at room temperature 25°C, with continuous agitation at 150 rpm on a gyratory shaker, for 48 h.

### Identification of PSB and phylogenetic analysis

#### DNA extraction

Bacterial cells were grown overnight at 28°C, in 100 mL Erlenmeyer flaks containing TSA medium (150 rpm). Cells were then harvested by centrifugation (10,000 *g*, 10 min at 4°C) and washed twice by centrifugation in saline solution. Washed cells were frozen in liquid nitrogen, lyophilized for 24 h, and resuspended in 1 mL of CTAB (2% hexadecyltrimethylammonium bromide, 1.4-M NaCl, 0.2% 2-mercaptoethanol, 20-mM EDTA, 100 mM Tris–HCl pH 8.0 (Sigma-Aldrich, Oakville, ON, Canada). Total genomic DNA was extracted using the FastDNA® SPIN Kit and FastPrep® Instruments (MP Biomedicals, Montreal, QC, Canada). Following extraction, the DNA was re-suspended in TE (10 mM Tris–HCl, 1 mM EDTA, pH 7.4), quantified using the spectrophotometer NanoDrop, and diluted in TE to give a concentration of 50 ng μL^−1^.

#### PCR amplifications

The gene encoding the 16S rRNA was amplified by the polymerase chain reaction (PCR) using the combination of universal primers pA (AGAGTTTGATCCTGGCTCAG) and pH (AAGGAGGTGATCCAGCCGCA) (Edwards et al., 1989). The PCR mixture consisted of deoxynucleotides at 200 μM each, 0.25 μM of each primer, 2.5 μM MgCl_2_, with 5 μL PCR buffer and 0.25 U of *Taq* DNA polymerase (5 Prime GmbH, Hamburg, Germany) (from (Taktek et al., 2015), with minor modifications). The following PCR conditions were used: 94°C for 2 min, followed by 30 cycles of 94°C for 30 s, 58°C for 30 s and 72°C for 1 min, and a final extension step at 72°C for 7 min.

#### Purification and sequencing of PCR products

The sizes of the PCR products were determined in 1% (W/V) Agarose TBE (Tris-Borate-EDTA, pH 8.3). The PCR products harboring an expected size of around 1,500 bp were used for further process.

The PCR purification and sequencing was performed by the Centre d’Expertise et de Service, Génome Québec (Montreal, QC, Canada). Sequences were identified using BLAST Nucleotide searches at NCBI website. All sequences were deposited in GenBank and their accession numbers are shown in Tables 1, 2.

**Table 1:** Taxonomic identification of rhizoplane isolates.

Root PSB isolates	Sample site	Seq. length (bp)	Identity (%)	Coverage (%)	Family	Species	Accession number
7*	L1-HighP	1,111	94	100	Pseudomonadaceae	*Pseudomonas mohnii*	OQ876719
8*	L1-HighP	991	91	100	Sphingomonadaceae	*Novosphingobium resinovorum*	OQ876703
19	L1-HighP	1,135	99	100	Pseudomonadaceae	*Pseudomonas* sp.	OQ876718
23	L1-HighP	989	99	100	Moraxellaceae	*Acinetobacter rhizosphaerae*	OQ876702
69	L1-LowP	714	99	100	Alcaligenaceae	*Achromobacter xylosoxidans*	OQ876701
82	L2-HighP	1,073	98	100	Pseudomonadaceae	*Pseudomonas azotoformans*	OQ876700
90	L2-HighP	942	99	100	Paenibacillaceae	*Paenibacillus xylanexedens*	OQ876716
92	L2-HighP	979	99	99	Pseudomonadaceae	*Pseudomonas* sp.	OQ876699
93	L2-HighP	988	98	99	Pseudomonadaceae	*Pseudomonas* sp.	OQ876714
96	L2-HighP	723	98	100	Paenibacillaceae	*Brevibacillus* sp.	OQ876713
99	L2-HighP	1,156	99	96	Paenibacillaceae	*Paenibacillus purispatii*	OQ876723
132	L2-LowP	1,097	99	100	Pseudomonadaceae	*Pseudomonas* sp.	OQ876722
133*	L2-LowP	857	89	99	Paenibacillaceae	*Paenibacillus amylolyticus*	OQ876721
136*	L2-LowP	1,193	95	99	Xanthomonadaceae	*Stenotrophomonas maltophilia*	OQ876736
146	L3-HighP	1,146	99	100	Bacillaceae	*Bacillus thuringiensis*	OQ876735
153*	L3-HighP	790	96	100	Pseudomonadaceae	*Pseudomonas* sp.	OQ876729
149	L3-HighP	1,060	99	100	Bacillaceae	*Bacillus nitratireducens*	OQ876734
164	L3-HighP	1,068	99	100	Pseudomonadaceae	*Pseudomonas fluorescens*	OQ876727
174	L3-HighP	983	99	100	Pseudomonadaceae	*Pseudomonas fluorescens*	OQ876720
185	L3-LowP	1,092	98	99	Pseudomonadaceae	*Pseudomonas* sp.	OQ876712
187*	L3-LowP	1,053	97	98	Paenibacillaceae	*Paenibacillus polymyxa*	OQ876711
196*	L3-LowP	826	96	100	Brevibacteriaceae	*Brevibacterium* sp.	OQ876710
202*	L3-LowP	577	96	100	Paenibacillaceae	*Brevibacillus* sp.	OQ876709
203	L3-LowP	1,025	99	100	Bacillaceae	*Bacillus* sp.	OQ876708

*Indicates isolates with a percentage of identity below 98%, which is the generally accepted threshold for sequence similarity at the species level for bacteria (Yarza et al., 2014). These strains require thorough characterization for taxonomic assignment and the potential establishment of a new species name.

**Table 2:** Plant growth promoting traits of rhizoplane bacterial isolates.

Rhizoplane PSB isolates	Species	Solubilization		Production					Fixation		Motility
		P^a^	P (μg mL^−1^)^b^	IAA	IAA (μg mL^−1^)^b^	NH_3_	Siderophore	Biofilm	N_2_	Flagella	Pili
7	*Pseudomonas mohnii*	(+ + +)	70.91 ± 2.17	(−)	20.60 ± 0.75	(+)	(−)	(−)	(−)	(+)	(−)
8	*Novosphingobium resinovorum*	(+ − −)	60.87 ± 0.77	(−)	6.82 ± 0.97	(+)	(−)	(−)	(+)	(+)	(−)
19	*Pseudomonadales* sp.	(+ + +)	70.01 ± 0.42	(+)	87.14 ± 0.84	(+)	(−)	(+)	(+)	(+)	(−)
23	*Acinetobacter rhizosphaerae*	(+ + +)	49.75 ± 0.33	(+)	48.85 ± 1	(+)	(−)	(−)	(+)	(+)	(−)
69	*Achromobacter xylosoxidans*	(+ + +)	82.56 ± 0.64	(−)	15.89 ± 2.05	(+)	(−)	(−)	(−)	(+)	(−)
82	*Pseudomonas azotoformans*	(+ + +)	39.13 ± 0.80	(+)	34.19 ± 3.01	(+)	(+)	(−)	(−)	(+)	(−)
90	*Paenibacillus xylanexedens*	(+ + +)	63.44 ± 0.66	(+)	69.56 ± 3.84	(+)	(−)	(−)	(+)	(+)	(+)
92	*Pseudomonas* sp.	(+ − −)	62.46 ± 0.03	(+)	51.80 ± 1.34	(+)	(−)	(−)	(−)	(+)	(−)
93	*Pseudomonas* sp.	(+ + +)	62.66 ± 0.02	(−)	11.52 ± 3.32	(+)	(−)	(−)	(−)	(+)	(−)
96	*Brevibacillus* sp.	(+ − −)	42.59 ± 0.45	(−)	6.15 ± 1.39	(+)	(−)	(−)	(−)	(+)	(−)
99	*Paenibacillus purispatii*	(+ − −)	260.01 ± 1.31	(−)	14.79 ± 1.55	(+)	(−)	(−)	(−)	(+)	(−)
132	*Pseudomonas* sp.	(+ + +)	65.07 ± 1.1	(+)	63.48 ± 1.33	(+)	(+)	(−)	(−)	(+)	(−)
133	*Paenibacillus amylolyticus*	(+ − −)	42.91 ± 0.45	(+)	330.27 ± 5.21	(+)	(−)	(−)	(−)	(+)	(−)
136	*Stenotrophomonas maltophilia*	(+ + +)	46.18 ± 0.87	(−)	10.14 ± 0.47	(+)	(−)	(−)	(−)	(+)	(−)
146	*Bacillus thuringiensis*	(+ + +)	63.64 ± 0.65	(+)	48.79 ± 2.41	(+)	(+)	(−)	(−)	(+)	(−)
153	*Pseudomonas* sp.	(+ + +)	68.66 ± 0.96	(+)	32.52 ± 0.8	(+)	(−)	(−)	(−)	(+)	(−)
149	*Bacillus nitratireducens*	(+ − −)	42.30 ± 0.29	(+)	53.30 ± 6.75	(+)	(−)	(−)	(−)	(+)	(−)
164	*Pseudomonas fluorescens*	(+ − −)	61.76 ± 0.54	(−)	3.23 ± 0.32	(+)	(−)	(−)	(−)	(+)	(−)
174	*Pseudomonas fluorescens*	(+ + +)	57.54 ± 0.6	(−)	7.25 ± 0.28	(+)	(−)	(−)	(−)	(+)	(−)
185	*Pseudomonas* sp.	(+ + +)	122.4 ± 0.44	(+)	39.90 ± 2.01	(+)	(−)	(+)	(−)	(+)	(−)
187	*Paenibacillus polymyxa*	(+ + +)	132.15 ± 0.21	(+)	251.58 ± 4.74	(+)	(−)	(−)	(+)	(+)	(−)
196	*Brevibacterium* sp.	(+ + −)	108.36 ± 0.9	(+)	242.03 ± 13.46	(+)	(−)	(−)	(−)	(+)	(−)
202	*Brevibacillus* sp.	(+ + +)	84.77 ± 0.11	(−)	10.35 ± 1.11	(+)	(−)	(−)	(−)	(+)	(−)
203	*Bacillus* sp.	(+ + +)	3.97 ± 0.06	(+)	35.50 ± 2.11	(+)	(+)	(−)	(−)	(+)	(−)

P, Phosphate solubilization test; IAA, Screening for Indole-3-acetic acid; NH_3_, Ammonia production; N_2_, Nitrogen fixation; (+): Positive for the trait; (−): Negative for the trait; (+ + +), (+ + −), and (+ − −): from strong to weak growth, respectively. ^a^Isolate’s responses after each of the three successive subcultures. ^b^Values are the mean of three replicates independent assays ± standard error.

### Further *in vitro* screening of PSB for additional plant growth promoting traits

All bacterial isolates that were identified as positive for phosphate solubilizing activity through at least, one subculture on NBRIP were referred to as PSB, as they possess the metabolic capability to solubilize phosphate. To further investigate them for different conventional plant growth promoting traits, all bioassays were performed three times for each strain.

#### Screening for Indole-3-acetic acid (IAA) and indole related compounds production

Qualitative and quantitative analysis of IAA and IAA-related compound production by PSB isolates was determined by spectrophotometry in culture medium supplemented with tryptophan as IAA-precursor, using the method of Salkowski (Biswas et al., 2018). Firstly, bacterial isolates were overnight cultured in 5 mL of LB medium. Then, 20 μL of bacterial suspension (standardized to OD 600 nm = 0.7) were inoculated into 15 mL Falcon tubes containing 5 mL 10% LB liquid supplemented with tryptophan (5 mM). The resulting cultures were incubated for 5 days at 28°C on a rotary shaker (150 rpm) and cells from culture were separated by centrifugation (10,000 *g*, 20 min, 4°C). Salkowski reagent [2% of 0.5 M ferric chloride (FeCl_3_) in 35% perchloric acid (HClO_4_)] were mixed with the culture supernatant (1/1 v/v) and the mixture was incubated in the dark at room temperature for 20 min. The development of a pink color indicating IAA production. Estimation of IAA production was performed spectrophotometrically at 530 nm using a standard IAA concentration curve prepared in LB 50% containing serial dilutions of the synthetic indol-3-acetic acid (Sigma-Aldric, Oakville, ON, Canada).

#### Siderophore production in agar medium

The chrome azurol S (CAS) assay, as described by Schwyn and Neilands (1987), was used to detect the siderophore production ability of PSB. In this colorimetric assay, siderophores sequester iron from the ternary complex CAS/iron (III)/hexadecyltrimethylammonium bromide (HDTMA), causing the release of the CAS dye and a consequent color change from blue to orange.

For CAS agar plate preparation, a liter of solution was prepared by dissolving 60.5 mg CAS in 50 mL glass-distilled water and combining it with 10 mL of iron (III) solution (1 mM FeCl_3_.6H_2_0 in 10 mM HCl). This mixture was added to 72.9 mg of HDTMA in 40 mL of distilled water. The resulting dark blue CAS solution was autoclaved for 15 min and then added to agar medium/Piperazine-1,4-bis (2-ethanesulfonic acid) (PIPES) mixture (30.24 g of PIPES in distilled water + salts solution +15 g of agar, pH adjusted to 6.8 using NaOH pellets before autoclaving) following the method by Louden et al. (2011).

Prior to inoculation, PSB isolates were cultured in 10% TSB (Tryptic Soy Broth, Becton Dickinson & Co., Franklin Lakes, NJ, USA) at 28°C for 48 h on a rotary shaker (150 rpm). The CAS plates were then spot-inoculated with PSB strains, with four bacterial strains per plate. The plates were observed for the development of an orange halo around the colonies after 7 days of incubation at 28°C. Each strain was tested in triplicate for both assays.

#### Ammonia production

Estimation of ammonia production by PSB isolates was carried out in qualitative assays with peptone as described in Dutta et al. (2015). Aliquots of fresh overnight culture of isolates in TSA were inoculated into 10 mL tubes of 10% peptone water (peptone 10 g L^−1^; NaCl 5 g L^−1^; distilled water 1 L), then incubated at 28°C for 72 h. After incubation, 0.5 mL Nessler’s reagent (10% HgI_2_, 7%KI; 50% aqueous solution of NAOH 32%) was added to each tube. Appearance of brown to yellow color indicated positive test for ammonia production (Marques et al., 2010).

#### Biofilm formation

Biofilm formation by isolates was assessed using the Crystal violet (CV) assay according to the method previously described by O'Toole (2011) with the following modifications proposed by Taktek et al. (2015). Inocula were prepared by growing bacteria overnight on a rotary shaker (180 rpm) at room temperature, in 25 mL of 10% TSB. Cells were collected and washed twice in 10 mL sterile saline (SS) (0.85% NaCl) after centrifugation (10,000 g, 5 min, 4°C) and re-suspended in SS. Flat bottom wells of sterile polystyrene 96-well microtiter plates (Costar, Corning Inc. Tewksbury, MA, USA) were filled with 100 μL of the modified NBRIP medium containing TCP. Then, each well was inoculated with 10 μL of a 48 h primary culture adjusted at an OD_600_ = 0.7. For these experiments, a non-inoculated TSB medium was used as the negative control. After 72 h of incubation at 28°C without agitation, the supernatant (i.e., medium and the non-adhering bacteria biofilm) were eliminated by simple inversion and each well was washed three times with 100 μL of sterile deionized water. The microplates were then air-dried for 30 min. The cells from the adhering biofilm were dyed with 100 μL of 0.1% crystal violet (CV) solution (30 min at room temperature). The excess of dye was removed by simple inversion and the biofilm was washed three times with running tap water at each wash. Then, the microplates were dried at room temperature during 10 min. Finally, bound CV was solubilized by adding 200 μL of 30% acetic acid solution and microtiter plates were incubated for 15 min. The absorbance in each well was sampled and the OD_530_ was read on a microplate reader. The results obtained were then transformed into a quantification of biofilm formation by calculating the average absorbance of three replicates and dividing by the average absorbance of the blank (without bacteria). The results expressed as this ratio can be considered as non-dependent from the non-specific coloring of the surface of the wells of the microplates by the CV. Following this procedure, biofilm formation was considered to have occurred when the ratio is greater than two (Brian-Jaisson et al., 2014).

#### In plate assays of nitrogen fixation

The Burks medium (HiMedia Laboratories, Kennett Square, PA, USA), containing inorganic salts along with carbon source but lacking nitrogen source, was used for detection of nitrogen fixing bacteria, able to fix nitrogen and grow when cultured on this nitrogen-free medium. Bacterial responses were observed after an incubation at 30°C for 7 days.

#### Twitching and swarming motility tests

Twitching motility is exclusively facilitated by type IV pili, with repetitive extension and retraction movements leading to the translocation of the cell body. This motility is observed on solid surfaces, interfaces, or in mediums with moderate viscosities (1% agar). Swarming motility, on the other hand, is mediated by flagella in collaboration with type IV pili and exhibits a dendritic pattern of movement. It is observed along semisolid surfaces, such as 0.3–0.7% agar, while *in vitro* conditions with 1.5% agar and low humidity inhibit the swarming form of motility (Otton et al., 2017).

Both motility assays were conducted following previous descriptions (De Kievit et al., 2001; Ochoa et al., 2015; Otton et al., 2017) with some modifications. Briefly, strains were cultured overnight in TSB at 37°C. For twitching motility assays, 1 μL of each bacterial suspension was stabbed into TSB agar (1%) to the bottom of the plate. The plates were then incubated at room temperature for 48 h, and the motility zone was observed. For flagellar swarming motility assays, TSB medium supplemented with 0.3% agar was prepared. Subsequently, 1 μL of each bacterial suspension was inoculated onto the agar, and the plates were incubated at 28°C. The diameters of the swarming zones were observed after 10 h. Each strain was tested in triplicate for both assays.

### Statistical analysis

To assess the normality of the data, the Shapiro test was conducted using XLSTAT (version 2022.2.1.1318). Subsequently, the Kruskal-Wallis non-parametric test, supplemented with a post-hoc Dunn test, was employed to identify significant differences among means at a 5% probability level (*p* ≤ 0.05). The experimental data provided represent mean values obtained from three replicates, presented as the mean ± standard error for each treatment.

## Results

### Identification of PSB and additional plant growth promoting traits

#### Rhizoplane PSB

A total of 210 isolates were obtained from root fragments collected from RP-rich soils in a mining area. From qualitative assays, 17% (35 isolates) of the isolates demonstrated clear TCP-solubilization halos on NBRIP solid medium, with diameters ranging from 0.5 to 4 cm. Through Sanger sequencing of the 16S rDNA, 24 PSB isolates were assigned to species using BLAST searches. These isolates were distributed among the bacterial phyla Pseudomonadota, Bacillota, and Actinomycetota (Table 1). Pseudomonadota and Bacillota dominated (with 14 and 9 isolates, respectively). Sequence comparisons of the 16S rDNA indicated similarities between 91 and 99% of known bacteria. Three families, Pseudomonadaceae, Bacillaceae, and Paenibacillaceae, dominated the bacterial community. Quantitative assays revealed varying P-solubilizing abilities, ranging from 39–260 μg mL^−1^ (Table 2). The highest value was observed for isolate 99, which was identified as *Paenibacillus purispatii*.

**Figure 2:**
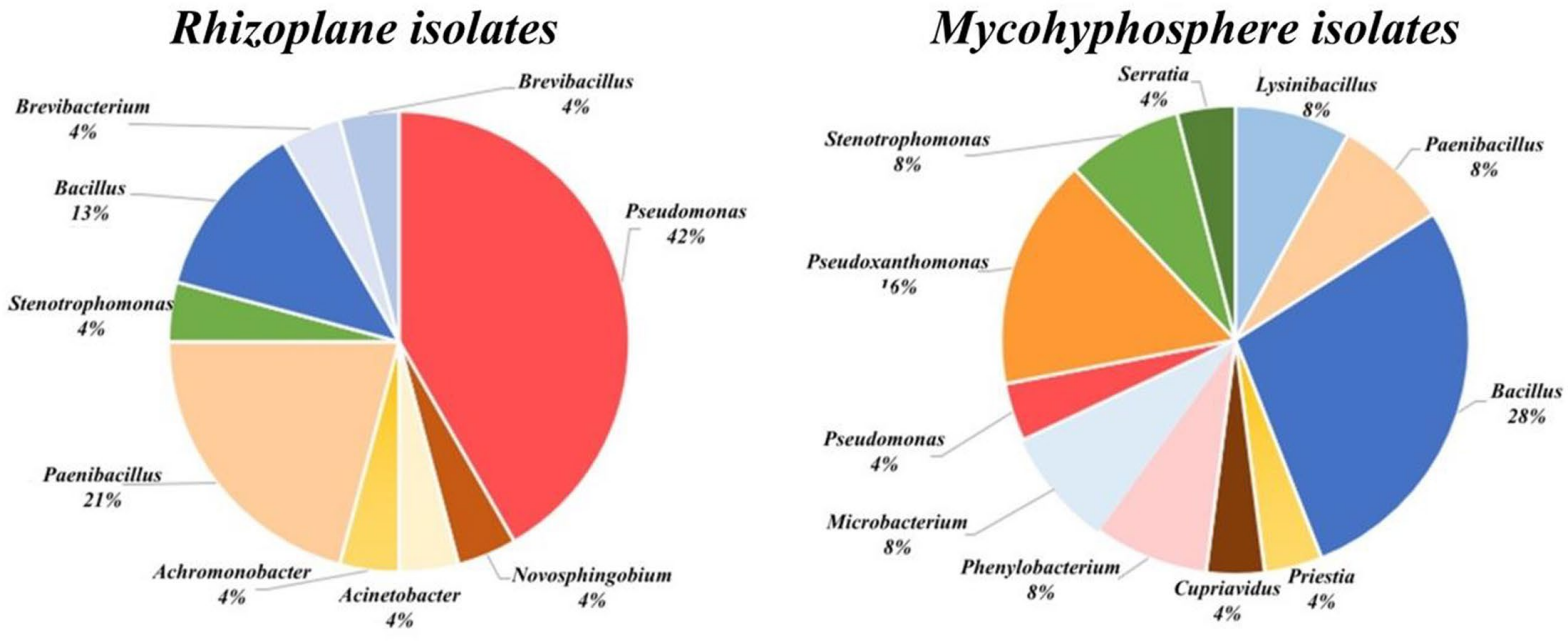
Taxonomic distribution of rhizoplane isolates (left panel) and mycohyphosphere isolates (right panel).

Most of the bacterial isolates were assigned to the Pseudomonadota and were affiliated to four genera: *Pseudomonas* (ten isolates), *Stenotrophomonas* (two isolates) *Acinetobacter* (one isolate). One isolate was affiliated with the Pseudomonadota and was associated to the genus *Achromobacter* with more 99% sequence identity. The remaining isolate fell within the α-Proteobacteria and Burkholderiales order, and it was identified as *Novosphingobium* (Figure 2).

Among Bacillota, the alignment of the sequences showed clustering of isolates with species of the genus *Paenibacillus* (four isolates), *Bacillus* (three isolates), or *Brevibacillus* (two isolates). The Actinomycetota phylum was represented with only one isolate affiliated within the Micrococcales as *Brevibacterium* species.

Phenotypic characteristics of the 24 isolates were assessed for different PGP traits (Table 2). Thus, all the isolates were positive for ammonia production. The IAA production was observed for 13 PSB isolates (Table 2). It ranged from 32.52 to 330.27 μg mL^−1^, with the isolates 133 (*Paenibacillus amylolyticus*), 187 (*P. polymyxa*) and 196 (*Brevibacterium*) showing the highest IAA production (330 ± 5.21, (Standard Error, SE), followed by 251.6 and 242 μg mL^−1^), respectively. Only five PSB were positively screened for N_2_ fixations (among them: one *Novosphingobium*, two *Paenibacillus*, one *Acinetobacter*), and four for siderophore capability (two isolates assigned to *Pseudomonas* and two as *Bacillus*) (Table 2). Two PSB among which one *Pseudomonas*, formed an important biofilm on an abiotic surface. The rhizoplane PSB mostly combined one additional PGP trait (nine isolates); six and eight PSB exerted, respectively, two and three additional capabilities. Phenotype of one PSB isolate (assigned to *Pseudomonas*) showed four additive PGP abilities (Table 2).

#### Mycohyphospheric PSB

From a collection of 44 mycohyphospheric isolates (Table 3), 25 isolates (56.8%) sampled from maize, leek, potato and tomato were identified as PSB from qualitative and quantitative TCP solubilization assays (Table 4). Nine isolates were obtained from maize plants, seven from tomato plants, five from leek plants, and four from potato plants. The isolates showed varying levels of P solubilizing activity ranging from 24.2 to 175.7 μg mL^−1^ and the isolate MI06 identified as *Paenibacillus typhae* exhibited the highest TCP solubilization activity (Table 4).

**Table 3:** Taxonomic identifications of mycohyphosphere isolates.

Hyphospheric PSB isolates	Sampled site	Seq. length (bp)	Identity (%)	Coverage (%)	Family	Species	Accession number
MI04	Maize/i	1,069	98%	98%	Xanthomonadaceae	*Pseudoxanthomonas* sp.	OQ876740
MI06	Maize/i	968	99%	100%	Bacillaceae	*Lysinibacillus sphaericus*	OQ876739
MI09	Maize/i	669	99%	100%	Yersiniaceae	*Serratia inhibens*	OQ876738
MI13	Maize/i	1,175	99%	100%	Bacillaceae	*Paenibacillus typhae*	OQ876737
MI14	Maize/i	1,028	99%	100%	Bacillaceae	*Paenibacillus xylanexedens*	OQ876733
MS03	Maize/s	1,083	99%	100%	Xanthomonadaceae	*Pseudoxanthomonas* sp.	OQ876732
MS09	Maize/s	851	99%	100%	Xanthomonadaceae	*Stenotrophomonas maltophilia*	OQ876730
MS11	Maize/s	1,055	99%	99%	Microbacteriaceae	*Microbacterium oxydans*	OQ876728
oMS04^#^	Maize/s	1,164	97%	99%	Xanthomonadaceae	*Stenotrophomonas maltophilia*	OQ876726
Ps*^#^	Leek/s	907	98%	100%	Bacillaceae	*Bacillus thuringiensis*	OQ876725
PS06	Leek/s	1,136	99%	100%	Bacillaceae	*Lysinibacillus fusiformis*	OQ876724
PS09	Leek/s	594	99%	100%	Xanthomonadaceae	*Pseudoxanthomonas* sp.	OQ876717
PS09b	Leek/s	590	99%	100%	Bacillaceae	*Bacillus wiedmannii*	OQ876715
Psi07	Leek/s	909	99%	99%	Caulobacteraceae	*Phenylobacterium* sp.	OQ876741
PTS08	Potatoe/s	829	99%	100%	Bacillaceae	*Bacillus thuringiensis*	OQ876743
TI01	Tomato/i	1,038	99%	100%	Bacillaceae	*Bacillus* sp.	OQ876707
TI04	Tomato/i	1,108	99%	98%	Caulobacteraceae	*Phenylobacterium* sp.	OQ876705
TI05^#^	Tomato/i	1,157	96%	100%	Xanthomonadaceae	*Pseudoxanthomonas* sp.	OQ876742
TS07	Tomato/s	1,037	99%	100%	Bacillaceae	*Bacillus* sp.	OQ876745
TS11	Tomato/s	1,079	99%	100%	Bacillaceae	*Bacillus toyonensis*	OQ876747
TS12	Tomato/s	1,138	98%	99%	Burkholderiaceae	*Cupriavidus necator*	OQ876706
TS18^#^	Tomato/s	1,120	98%	100%	Microbacteriaceae	*Microbacterium* sp.	OQ876704
PTS17	Potatoe/s	870	99%	100%	Bacillaceae	*Bacillus thuringiensis*	OQ876731
PTS06^#^	Potatoe/s	857	98%	100%	Pseudomonadaceae	*Pseudomonas* sp.	OQ876746
PTS03^#^	Potatoe/s	767	97%	100%	Bacillaceae	*Priestia megaterium*	OQ876744

(i): Plant supplemented with igneous RP; (s): Plant supplemented with sedimentary RP. ^#^Indicates isolates with a percentage of identity below 98%, which is the generally accepted threshold for sequence similarity at the species level for bacteria (Yarza et al., 2014). These strains require thorough characterization for taxonomic assignment.

**Table 4:** Plant growth promoting traits of mycohyphosphere isolates.

Mycoyphospheric PSB isolates	Species	Solubilization		Production					Fixation	Motility	
		P^a^	P (μg mL^−1^)^b^	IAA	IAA (μg mL^−1^)^b^	NH_3_	Siderophore	Biofilm	N_2_	Flagella	Pili
MI04	*Pseudoxanthomonas* sp.	(+ + +)	59.46 ± 0.08	(+)	76.59 ± 6.5	(+)	(−)	(−)	(+)	(+)	(−)
MI06	*Lysinibacillus sphaericus*	(+ + +)	175.69 ± 0.61	(−)	7.61 ± 0.29	(+)	(−)	(−)	(−)	(+)	(−)
MI13	*Paenibacillus typhae*	(+ + +)	151.99 ± 0.22	(+)	566.67 ± 2.61	(+)	(−)	(−)	(−)	(+)	(−)
Mi14	*Paenibacillus xylanexedens*	(+ + +)	65.23 ± 0.25	(+)	963.96 ± 8.05	(+)	(−)	(−)	(+)	(+)	(−)
MS03	*Pseudoxanthomonas* sp.	(+ + +)	54.76 ± 0.22	(+)	139.11 ± 2.47	(+)	(−)	(−)	(−)	(+)	(+)
MS09	*Stenotrophomonas maltophilia*	(+ + +)	98.28 ± 3.17	(−)	3.96 ± 0.53	(+)	(−)	(−)	(+)	(+)	(−)
MS11	*Microbacterium oxydans*	(+ − −)	166.44 ± 0.17	(+)	73.27 ± 0.96	(+)	(−)	(−)	(+)	(+)	(−)
oMS04	*Stenotrophomonas maltophilia*	(+ + +)	73.95 ± 0.09	(+)	126.32 ± 1.13	(+)	(−)	(−)	(+)	(+)	(+)
Ps*	*Bacillus thuringiensis*	(+ + −)	73.28 ± 0.30	(+)	60.3 ± 3.74	(+)	(−)	(−)	(+)	(+)	(−)
PS06	*Lysinibacillus fusiformis*	(+ − −)	79.97 ± 0.37	(+)	110.83 ± 2.93	(+)	(−)	(+)	(−)	(+)	(−)
PS09	*Pseudoxanthomonas* sp.	(+ + +)	64.16 ± 0.61	(+)	88.74 ± 6.83	(+)	(−)	(−)	(−)	(+)	(−)
PS09b	*Bacillus wiedmannii*	(+ + −)	67.41 ± 0.50	(+)	46.75 ± 2.62	(+)	(−)	(−)	(−)	(+)	(−)
Psi07	*Phenylobacterium* sp.	(+ + +)	51.92 ± 0.53	(+)	80.21 ± 4.83	(+)	(−)	(−)	(−)	(+)	(−)
PTS08	*Bacillus thuringiensis*	(+ + +)	24.15 ± 0.08	(+)	425.97 ± 5.85	(+)	(−)	(−)	(−)	(+)	(−)
TI01	*Bacillus* sp.	(+ + +)	64.73 ± 0.70	(+)	97.51 ± 1.16	(+)	(−)	(−)	(−)	(+)	(−)
TI04	*Phenylobacterium* sp.	(+ + −)	81.94 ± 0.36	(+)	107.09 ± 2.15	(+)	(−)	(−)	(+)	(+)	(−)
TI05	*Pseudoxanthomonas* sp.	(+ + −)	48.23 ± 0.22	(−)	18.41 ± 0.95	(+)	(−)	(+)	(+)	(+)	(−)
TS07	*Bacillus* sp.	(+ + +)	45.09 ± 0.15	(+)	49.74 ± 3.74	(+)	(−)	(−)	(+)	(+)	(−)
TS11	*Bacillus toyonensis*	(+ + +)	41.56 ± 0.22	(+)	51.5 ± 1.15	(+)	(−)	(−)	(+)	(+)	(−)
TS12	*Cupriavidus necator*	(+ + +)	133.59 ± 0.83	(−)	6.85 ± 2.37	(+)	(−)	(+)	(+)	(+)	(−)
TS18	*Microbacterium* sp.	(+ + −)	99.09 ± 0.32	(+)	75.98 ± 1.38	(+)	(−)	(−)	(+)	(+)	(−)
PTS17	*Bacillus thuringiensis*	(+ + +)	44.68 ± 0.07	(+)	41.40 ± 2.30	(+)	(−)	(−)	(−)	(+)	(−)
PTS06	*Pseudomonas* sp.	(+ + −)	64.10 ± 0.77	(−)	21.12 ± 3.59	(+)	(−)	(+)	(−)	(+)	(−)
PTS03	*Priestia megaterium*	(+ + +)	56.44 ± 0.77	(+)	128.2 ± 0.74	(+)	(−)	(−)	(−)	(+)	(−)
MI09	*Serratia inhibens*	(+ + −)	170.05 ± 0.24	(+)	246.7 ± 8.69	(+)	(−)	(+)	(+)	(+)	(+)

P, Phosphate solubilization test; IAA, Screening for Indole-3-acetic acid; NH_3_, Ammonia production; N_2_, Nitrogen fixation; (+): Positive for the trait; (−): Negative for the trait; (+ + +), (+ + −), and (+ − −): from strong to weak growth, respectively. ^a^Isolate’s responses after each of the three successive subcultures. ^b^Values are the mean of three replicates independent assays ± standard error.

Of the selected PSB, 11 were affiliated to the Pseudomonadota phylum as shown in Table 3, seven in the Campylobacterota phylum with isolates displaying sequence identities with *Pseudoxanthomonas* (five isolates) or *Stenotrophomonas* (two isolates) (Figure 2). One strain was identified as β-Proteobacteria and Burkholderiales, close to the referenced *Cupriavidus necator*. In addition, two isolates grouped within the α-Proteobacteria class, related to Caulobacteraceae family and *Phenylobacterium* sp.

In addition, 12 isolates were affiliated to Bacillota, all being assigned to the Bacilli class. These isolates were mainly related to *Bacillus* (seven isolates), PTS08 sharing more than 99.5% identity with *Bacillus*. The other ones shared sequence identity with representatives of *Paenibacillus (two)*, *Priestia* (one) or *Lysinibacillus* (two) genus. The Actinomycetota phylum is represented with two *Microbacterium* isolates, one of which is related to *Microbacterium oxydans*.

Among the 25 mycohyphospheric PSB selected, all were identified as ammonia producers, while 20 isolates representing nine genera (with *Bacillus* being prevalent) displayed IAA production ranging from 41.4 to 963.9 μg mL^−1^ (Table 4). The isolates MI14 (*P. xylanexedens*), MI13 (*P. typhae*) and PTS08-(*B. thuringiensis*) exhibited the highest IAA production (963.9, 566.6, and 425.9 μg mL^−1^, respectively). Only 12 isolates showed nitrogen fixation, belonging to seven different genera. No isolate produced siderophore. Four isolates belonging to *Pseudoxanthomonas* (two isolates), *Cupriavidus* (one isolate), and *Lysinibacillus* (one isolate) exhibited biofilm formation.

All rhizoplane and mycohyphospheric isolates were found to be positive for swarming motility due to flagella. Pili motility was observed for one rhizoplane isolate (*Paenibacillus*) and three mycohyphospheric isolates assigned to *Stenotrophomonas*, *Pseudoxanthomonas* (Tables 3, 4).

### Distinct taxonomic profiles among PBS communities captured from the rhizoplane and mycohyphosphere samples

Interestingly, the two experimental setups produced distinct taxonomic profiles of PSBs, revealing both shared and unique taxa at the genus and species levels. Combining Sanger sequencing and phylogenetic analysis revealed that isolates from the rhizoplane and mycohyphospheric environments belonged to only three bacterial phyla: Pseudomonadota, Bacillota, and Actinomycetota (Tables 1, 3).

Four genera (*Bacillus*, *Paenibacillus*, *Pseudomonas*, and *Stenotrophomonas*) were common to both bacterial collections, with isolates related to Bacillus and Paenibacillus (Bacillota) prevalent in both environments. In contrast, *Pseudomonas* and *Pseudoxanthomonas* were dominant in the rhizoplane and mycohyphosphere, respectively (Figure 2).

At the species level, five species (17%) were shared, namely *Bacillus thuringiensis*, *Paenibacillus xylanexedens*, Pseudomonas sp., Bacillus sp., and *Stenotrophomonas maltophilia* (Tables 2, 4). Isolates related to Microbacteriaceae, Caulobacteraceae, Burkholderiaceae, and Yersiniaceae were specifically identified among the mycohyphospheric isolate collection, displaying the largest taxonomic diversity at the family level. A representative of Moraxellaceae was exclusively identified among rhizoplane isolates.

## Discussion

### Prospecting PSB in little-explored habitats from RP-enriched environments

This study explores the potential of PSBs as bioinoculants to enhance the efficiency of low-solubility RP ore used directly in some countries as a cost-effective phosphate fertilizer in soils. Despite the widespread use of microbial-based P fertilizers, discrepancies in efficacy exist in the literature, likely due to environmentally and context-dependent factors influenced by complex interactions between soil P chemistry and microbiology (Ducousso-Detrez et al., 2022a). The study emphasizes the need for more mechanistic data and advocates actively developing new inoculants for diverse environmental conditions, selecting strains with high field success and minimal downstream impact. It suggests exploring unique or extreme habitats for prospecting novel microbial diversity. To achieve this goal, two distinct experimental setups were designed to isolate PSBs from specific environments enriched with RP. These setups targeted rhizoplane habitats within mining environments and mycohyphospheric habitats, both of which hold ecological significance.

The second experimental setup focused on the specificity of hyphosphere habitats. Studies suggest that AMF hyphae recruit their own soil microbiomes in their hyphosphere, distinct from those of the bulk soil or rhizosphere (Wang G. et al., 2023; Wang L. et al., 2023). However, the diversity, richness, and structure of hyphosphere microbiomes are only beginning to be understood (Emmett et al., 2021; Zhang et al., 2022; Basiru et al., 2023). Soil microbes colonizing the hyphosphere hold significant interest due to their potential functions, likely to contribute to or complement the specific functional capabilities of AMF in a particular context (Faghihinia et al., 2022; Zhang et al., 2022; Wang G. et al., 2023; Wang L. et al., 2023).

The last step (in bi-compartmented Petri dishes) was conducted to select AMF-associated bacteria growing along the hyphae of *R. irregularis*, i.e., mycohyphospheric bacteria from agronomic soils.

These two setups proved to be effective strategies to select PSB isolates. Particularly, our experimental setup confirms that two-compartment Petri dishes are a useful tool to target mycohyphospheric PSB, aligning with previous reports (Ordonez et al., 2016; Taktek et al., 2017).

Using the TCP assay, which remains prevalent in current literature despite being considered relatively weak for studying P solubilization capabilities of PSBs (Bashan et al., 2013), some root and mycohyphosphere isolates demonstrated promising results. They released solubilized P concentrations up to 260 and 175.6 μg mL^−1^ in 7 days, respectively. In light of these findings, PSBs with high phosphate-solubilizing potential could be relevant as potential compounds for inoculant engineering and subsequent use in combination with low-availability P forms such as RP to promote plant growth.

### Rhizoplane PSB in RP-rich soils with high P availability from the mining area

Around 16% of the initially sampled rhizoplane isolates were identified as culturable PSB. According to Khan et al. (2007), phosphate-solubilizing microorganisms (Fungi and Bacteria) could make up to 50% of culturable populations, and their presence, abundance, and diversity are likely influenced by the plant species. Similarly, according to Goswami et al. (2016), estimates range from 20 to 40%. The differences in percentages observed in various studies may be attributed to various factors, including the selection of culture techniques. Notably, the selection and cultivation of PSB can be carried out from different steps of the *in vitro* culture process and performed on different culture media (Pikovskaya, 1948; Nautiyal, 1999), potentially favoring some taxa over others. Furthermore, it has been found that the rhizosphere contains a higher proportion of PSBs than the surrounding root-free soil (Mander et al., 2012; Nicolitch et al., 2016). Extensively, Spohn et al. (2020) established the relative abundance of PSB to be 24.4% in the rhizoplane (root surface), which was significantly higher than in the rhizosphere or on saprolite (a chemically weathered bedrock) where plants were growing (9.5 and 2.2%, respectively). This ecological data can obviously impact the percentage of the cultivable fraction among soil PSB. Nevertheless, many questions remain unanswered regarding the achievement of a consensus allowing more rigorous comparison of PSB studies in a relative manner. The choice of culture-dependent techniques, the development of culture methods for a larger number of bacterial taxa, the standardization of protocols (from sampling design to characterization of isolates and their sequencing, the comprehensive description of soil microbiomes, notably to further characterize the relation between taxonomy and functions), are still major challenges for the future of microbiome research and barriers to the replication of the same sampling strategies in different environments by different researchers. In a comparative approach, Stefani et al. (2015) employed both culture-independent methods, such as amplicon sequencing of soil DNA, and culture-dependent techniques to characterize bacterial communities in hydrocarbon-contaminated soils. Their findings indicated that these methods captured distinct microbial community fractions in soils. Notably, a significant proportion of taxa identified through culture-independent methods, including many of the most abundant taxa *in situ*, remained unrecovered by culture, despite the utilization of various media. Moreover, they observed only a 2% increase in bacterial species richness when isolation techniques were applied. These results highlight the fragmented nature of our understanding of soil microbial diversity, primarily due to biases introduced by the methods employed (for a comprehensive review, refer to Basiru et al., 2023). They underscore the need for ongoing efforts to explore and disentangle this diversity.

Interestingly, Spohn et al. (2020) also showed enrichment of PSB in the rhizosphere of plants grown on saprolites with low P availability compared to saprolites with a higher soluble P fraction. In light of these results, it is important to note that in our work, a significant proportion of PSB were successfully screened from the RP-rich soils where P availability was high (up to 339.5 mg kg^−1^ P Olsen). Here, our results raise some questions for the future: are the mechanisms classically associated with P solubilization and on which the *in vitro* selection of PSB is based (i.e., the release of organic acids) really contribute to soil P solubilization and to the high level of orthophosphate ions in the mining soils with elevated available P levels? Or do *in situ* PSB occurrences link to other functionalities in these soils [e.g., physicochemical mechanisms behind the phosphate solubilization trait could be involved in iron solubilization; indeed, in aerobic conditions and neutral pH, Fe is almost insoluble for plants (Schwab and Lindsay, 1983)]

Interestingly, a higher proportion of mycohyphospheric PSB was obtained compared to rhizoplane PSB (57 and 11% respectively). This outcome is notable, considering that the selection process for mycohyphospheric isolates could have significantly reduced the probability of selecting PSB isolates due to the multiple steps in the procedure and their inherent selectivity. Consequently, one could infer that the mycohyphospheric PSB isolation protocol is more efficient than the one for rhizoplane PSB isolation, particularly in terms of the proportion of PSB within the collection of cultivable PSB isolates. The differences in the quality and quantity of exudates from plant roots and AMF extraradical hyphae could differentially contribute to microbial community dynamics, symbiotic associations, soil structure, and chemical signaling in the rhizosphere. Ultimately, these factors may influence the isolation methods of microorganisms from these two biotopes (Zhang et al., 2022; Basiru et al., 2023).

### Diversity of culturable root and mycohyphospheric PSB isolated from RP-rich habitats

In this study, representatives of Pseudomonadota, Actinomycetota and Bacillota are dominant. Such data are in accordance with previously published results. Thus, Pseudomonadota, Actinomycetota, and, to a lesser extent, Bacillota have been described as ubiquitous in various soils worldwide (Delgado-Baquerizo et al., 2018). Besides, the taxonomic composition of bacterial communities from alkaline phosphate mine wastes showed that sequences belonging to Pseudomonadota and Actinomycetota, as well as the genera *Pseudomonas* and *Bacillus* were highly represented (Mghazli et al., 2021). Similarly, Pseudomonadota dominated the root-associated soils and bulk soil associated with Chinese mining sites (Ye et al., 2020). Furthermore, these three phyla are well-known in the literature to include PGPR and effective P-solubilizers (Mghazli et al., 2021). The growth-promoting abilities of *Pseudomonas* and *Bacillus* genera, which are widespread in various soils worldwide, are well documented and have been found to be effective and abundant P-solubilizers (Sharma et al., 2014; Kumar et al., 2016; Vacheron et al., 2016). Therefore, some of them are incorporated into the formulation of diverse microbial biofertilizers, available on the international market for farmers (Goswami et al., 2016; Kumari et al., 2019; Lobo et al., 2019). These findings are in accordance with the data of Yang et al. (2012), who classified 123 PSB, isolated from P-rich soils from a lake drainage area, into three bacterial phyla: Pseudomonadota (with some *Pseudomonas* representatives), Actinomycetota, and Bacillota (including *Bacillus* and *Brevibacillus*).

However, we must highlight an under-representation of Actinomycetota; only three Actinomycetota isolates were identified and referred to as genera *Microbacterium* (two mycohyphospheric isolates) or *Brevibacterium* (one root isolate). This phylum is ubiquitously distributed in a large range of soils (Pierzynski et al., 2005; Qin et al., 2016). Moreover, a large number of Actinomycetota exhibit PGP traits, with several being P-solubilizers (Hamedi and Mohammadipanah, 2015), either as free-living bacteria or endophytic bacteria (Qin et al., 2017; Chen et al., 2019). Similarly, Yang et al. (2012) only identified three isolates as Actinomycetota among the 123 PSB they selected. It is likely due to the culture-dependent procedures which were not optimal for Actinomycetota. For example, the effectiveness of TCP-based medium for assessing the *in vitro* capability of bacteria for solubilization may be suboptimal in certain taxa (Bashan et al., 2013, 2014).

The collection also comprises isolates associated with less common taxa (e.g., Microbacteriaceae, Caulobacteraceae, Burkholderiaceae, Yersimiaceae), expanding our understanding of PSB in RP-rich environments. These promising results are expected to contribute to the advancement of environmentally friendly fertilization practices compared to current methods.

### Multifunctionality of PSB isolates for inoculant formulation

Our study demonstrated that the selected PSB possessed additional and diverse PGP abilities. Ammonia production emerged as the most prevalent PGP trait among the isolates, with all tested isolates showing a positive result. This trait is acknowledged for its role in enhancing plant growth by providing available nitrogen to the host plant, thereby promoting root and shoot elongation (Hayat et al., 2010; Kandjimi et al., 2015). Furthermore, approximately 58.3 and 80% of rhizoplane and mycohyphospheric isolates, respectively, demonstrated the ability to produce IAA after incubation with tryptophan as the auxin precursor. IAA production is associated with increased root elongation, lateral root formation, and root hairs, potentially enhancing the efficiency of the plant’s root system for water and nutrient uptake (Jain and Patriquin, 1985). Consistent with these findings, some reports suggest that PSB may exert a more significant effect on root traits (such as root biomass, diameter, length, surface, or volume) than rhizosphere phosphate solubilization alone (Elhaissoufi et al., 2020), highlighting the importance of selecting isolates with both IAA production and PSB traits.

Notably, all isolated bacteria exhibited motility, adding another interesting feature likely to facilitate rhizoplane colonization.

## Conclusion

Addressing P deficiency in croplands, especially those utilizing phosphate-based fertilizers, has spurred interest in microorganisms capable of converting insoluble P into bioavailable forms. We conducted two independent experiments in distinct habitats to capture PSBs, resulting in diverse PSB communities. These findings, demonstrate the efficacy of both protocols in selecting a broader cultivable PSB biodiversity, and hold promise for more environmentally friendly fertilization practices. Future investigations should incorporate diverse cultivation media and techniques to reduce potential biases and capture bacterial diversity closer to that observed in natural habitats (Chen and Liu, 2019). Additionally, investigations on bacterial taxonomic diversity, community structure, and functions in the mining area [using sequencing and phylogenetic analysis of the 16S rRNA gene combined with OMICS technologies (Mghazli et al., 2021)] would be relevant for identifying taxa and functions involved in P cycling in native P mining environments.

Also, further greenhouse and field studies will be essential in the future to test the dual use of mineral nutrient and microbial resources (individually or in polymicrobial formulations, multi-species, and pluri-functional) for agronomic purposes. Exploring PSB from P-rich soils could aid in developing sustainable microbe-assisted rehabilitation strategies for derelict phosphate mine lands. Our data may enhance phytoextraction strategies, utilizing efficient soluble-P producers, or phytoremediation efforts in phosphate mining wasteland soils. Further research into the molecular mechanisms of PSB evolution and adaptation to complex environments, such as RP-rich mining soils, is a pertinent avenue for exploration.

## Data availability statement

The accession numbers for the 16S rDNA sequences of all isolates utilized in this study are presented in Tables 1, 3 for rhizoplane and mycohyphosphere isolates, respectively.

## Author contributions

AD-D: Formal analysis, Methodology, Writing – original draft. ZL: Methodology, Writing – review & editing. JF: Supervision, Writing – review & editing. AL-H: Conceptualization, Funding acquisition, Supervision, Writing – review & editing. MH: Conceptualization, Funding acquisition, Project administration, Supervision, Writing – review & editing.
